# Nudging New York: adaptive models and the limits of behavioral interventions to reduce no-shows and health inequalities

**DOI:** 10.1186/s12913-020-05097-6

**Published:** 2020-04-26

**Authors:** Kai Ruggeri, Tomas Folke, Amel Benzerga, Sanne Verra, Clara Büttner, Viktoria Steinbeck, Susan Yee, Krisda Chaiyachati

**Affiliations:** 1 Department of Health Policy and Management, Columbia University Mailman School of Public Health, 722 W 168th St., New York, NY 10032 USA; 2 Faculty of Health, Medicine and Life Science, Maastricht University, Universiteitssingel 40, 6229 ER Maastricht, The Netherlands; 3 Community Healthcare Network, New York City, 60 Madison Ave., New York, NY 10010 USA; 4 Perelman School of Medicine, University of Pennsylvania, 423 Guardian Drive, Blockley Hall Room 1313, Philadelphia, PA 19104 USA

**Keywords:** No-shows, Healthcare, Low-income populations, Economic inequality, Health inequality, FQHC, Personalized interventions, Behavioral interventions, Overbooking

## Abstract

**Background:**

Missed healthcare appointments (no-shows) are costly and operationally inefficient for health systems. No-show rates are particularly high for vulnerable populations, even though these populations often require additional care. Few studies on no-show behavior or potential interventions exist specifically for Federally Qualified Health Centers (FQHCs), which care for over 24 million disadvantaged individuals in the United States. The purpose of this study is to identify predictors of no-show behavior and to analyze the effects of a reminder intervention in urban FQHCs in order to design effective policy solutions to a protracted issue in healthcare.

**Methods:**

This is a retrospective observational study using electronic medical record data from 11 facilities belonging to a New York City-based FQHC network between June 2017 to April 2018. This data includes 53,149 visits for 41,495 unique patients. Seven hierarchical generalized linear models and generalized additive models were used to predict no-shows, and multiple regression models evaluated the effectiveness of a reminder. All analyses were conducted in R.

**Results:**

The strongest predictor of no-show rates in FQHCs is whether or not patients are assigned to empaneled providers (z = − 91.45, *p* < 10^− 10^), followed by lead time for appointments (z = 23.87, p < 10^− 10^). These effects were fairly stable across facilities. The reminder had minimal effects on no-show rates overall (No show rate before: 41.6%, after: 42.1%). For individuals with appointments before and after the reminder, there was a small decrease in no-shows of 2%.

**Conclusions:**

The limited effects of the reminder intervention suggest the need for more personalized behavioral interventions to reduce no-shows. We recommend that these begin with increasing the use of empaneled providers for preventive care appointments and reducing the lag time between setting the appointment and the actual date of the appointment, at least for individuals with a high rate of no-show. By complementing these with low-intensity, low-cost behavioral interventions, we would expect greater impacts for improved access to care, contributing to the well-being of vulnerable populations.

## Background

Missed healthcare appointments are costly and operationally inefficient for health systems [1]. It is estimated that patients miss (no-show) as many as 23.5% of all medical appointments in North America [2]. People with lower socioeconomic status are more likely to miss medical appointments [3], but are also likely to require more outpatient care [4]. As a result, no-shows can directly accelerate inequalities in life expectancy and well-being [5].

In the United States, Federally Qualified Health Centers (FQHCs) are a key provider of safety-net services for over 24 million low-income people who otherwise might not receive care [6]. FQHCs typically provide care for disadvantaged populations in underserved areas at little or no direct cost to patients, with a large number of patients qualifying for Medicare/Medicaid. Given the at-risk population they serve, access to preventive care is especially critical in FQHCs, which must provide care regardless of patients’ ability to pay.

Unfortunately, no-show rates at FQHCs can be as high as 45% [7]. As FQHCs serve a high-need population with limited resources, high no-show rates are believed to lead to inefficiency and to create a serious financial strain on healthcare systems. Therefore, high no-show rates test the financial solvency of FQHCs and endanger future access to care for those with the greatest need.

Low-income populations face profound personal, social, economic, and political barriers to primary care practices and preventive care [8]. These barriers include a lack of transportation [9], miscommunications [3], anxiety [9], forgetting the appointment [3], a lack of respect perceived in treatment [9], and a lack of understanding of the (financial) impact of a no-show [9]. Given these barriers, there is a clear space for behavioral interventions to support attendance for those that might wish to attend. Specifically, this could include interventions that apply principles from nudging, which involve small changes in the choice architecture that encourage optimal choices while ensuring autonomy remains with individuals, utilizing theory from behavioral economics and social psychology [10].

Due to low costs, simple behavioral interventions such as nudges are an appealing option for implementing at FQHCs [11]. One commonly used nudge to reduce no-show is a reminder in the form of text messages and phone calls (including automated "robocalls") typically sent 7 days to 24 hours before a scheduled appointment [12, 13]. While some effects have been found for reminders [14], there is limited information about their value specifically in low-income settings [15].

This study aims to identify predictors of no-shows, and to examine the effectiveness of the reminder for urban, low-income patients. These insights contribute to the identification and development of effective measures to reduce no-shows. Interventions to reduce no-shows in this way will not only contribute to more efficient delivery of healthcare services to disadvantaged populations, but also directly benefit the health of the patients they serve.

We utilized data from an urban FQHC in New York City as it is facing a high rate of no-shows while attempting to serve an especially at-risk population. It is also a useful study population given it has multiple clinics positioned across four of the five boroughs (all but Staten Island) and was in the process of trialing multiple interventions to increase attendance in the window of data used. We focused on these data as a useful indicator of urban FQHCs, but wider study including FQHCs in rural areas would add further insight. To best model the data available, a dynamic analytical approach was necessary, including descriptive and adaptive predictive models to best understand behaviors across this population.

## Methods

### Setting

Only no-show data for preventive appointments are included in this study, as failing to attend for treatment or urgent care requires extensive consideration of additional psychological and structural factors. Preventive appointments offered at this setting include initial or annual appointments for lifestyle advice, family practice, gynecology, as well as infectious disease. The reminder interventions includes a robocall delivered 3 days in advance, followed up by a text message reminder 2 days before the appointment. This reminder intervention aimed to nudge participants to attend their scheduled preventive appointments.

### Data

This is a retrospective observational study using electronic medical record data from 11 facilities belonging to a New York City-based FQHC network. This network provides care for an ethnically diverse, underserved population. Data were collected between June 15, 2017, and April 30, 2018. The initial dataset included 65,410 patients. After the cleaning process, which eliminated duplicative data strings with the same encounter ID and other problematic cases, the total number of patients identified was 63,842. To avoid other forms of duplication and ambiguous cases, predictive analyses were run on a total of 53,149 visits for 41,495 unique patients. For all 11 sites, data collection started prior to the implementation of a reminder intervention. Clinics began interventions in waves of implementation, resulting in the removal of 2 weeks of data due to the ambiguity in the timing of rollout. The analysis on the predictors of no-show and the effectiveness of the reminder were based on 53,149 appointments. Additionally, analyses on the effectiveness of the reminder have been conducted including only the 5569 individuals who had appointments scheduled both before and after the reminder was implemented.

### Prediction of no-show analysis

To build models of behavior based on key predictors, no-shows were the main dependent variable in seven statistical models. Predictors of no-show behavior were identified based on the borough of patient residence, the healthcare center they attended, whether the visit was with their primary (empaneled) healthcare provider, and lead time (the time between when the appointment was made and when it was due to take place). Interactions between the independent variables were also tested. Because patients were clustered in boroughs and healthcare centers, hierarchical generalized linear models were used to evaluate these relationships, as well as generalized additive models to test for non-linear effects. As the outcomes are binary (i.e., attend or no-show), these models are binomial with a logistic link function.

The seven statistical models of increasing complexity were compared against a null-model predicting every person would attend their appointment (see Table 1 for descriptions of all models). The performance of each model was determined by using a five-fold cross validation, meaning that each model has been evaluated based on how well it could predict unobserved data.

**Table 1:** The Candidate Models to Predict no-show rate across healthcare centers

*Model*	*Description*
*0*	*Predicting no-show status from a central intercept term, will always predict the most common outcome.*
*1*	*Allows the intercept to vary by facility and borough.*
*2*	*Allows the intercept to vary by facility and borough. Lead Time and Empaneled Provider have the same linear effect across all facilities and boroughs.*
*3*	*Allows the intercept to vary by facility and borough. Lead Time and Empaneled Provider can have different linear effect across all facilities and boroughs.*
*4*	*Allows the intercept to vary by facility and borough. Lead Time and Empaneled Provider have the same non-linear effects across all facilities and boroughs.*
*5*	*Allows the intercept to vary by facility and borough. Lead Time and Empaneled Provider can interact, so that the non-linear effect of lead time depends on whether respondents are visiting their empaneled provider or not.*
*6*	*Allows the intercept to vary by facility and borough. Empaneled Provider has the same effect everywhere, but lead time can have different non-linear effects in different facilities, but are shrunk towards a central, non-linear function.*
*7*	*Allows the intercept to vary by facility and borough. Empaneled Provider has the same effect everywhere, but lead time can have different non-linear effects in different facilities, without any constraints on variations between facilities.*

Classification accuracy and Gini coefficients were the measures of fit used in the predictive modeling. Classification accuracy is the proportion of test cases correctly classified by the model. In this case, this means the proportion of the number of times a no-show was predicted and a no-show actually occurred. Classification accuracy is the fundamental outcome of interest. However, it is not the most sensitive measure for model comparisons. Gini coefficients derived from signal detection theory provide a more sensitive metric for predicting binary outcomes. Chance performance results in a coefficient of 0 and perfect discrimination provides a coefficient of 1. Gini coefficients are directly linked to AUROC so that GINI = 2*AUROC – 1. The null-model has a Gini coefficient of 0 because the null-model always makes the same prediction, regardless of the outcome. For further details about this analysis see Appendix A.

### Intervention effectiveness analysis

To examine the effectiveness of the reminder intervention, a hierarchical generalized linear model tested the effect of the intervention for the full sample. In addition, a chi-square test was conducted on the 5569 patients with appointments before and after the intervention, and a hierarchical model tested whether the effect of the reminders was moderated by lead time. The latter was conducted to identify if there was an interval in lead time during which the reminders were particularly effective.

## Results

### Participants

Overall, the sample was 41% Hispanic. The sample included African American (37%), White (12%), and Asian (4%) patients, although 45% did not report race. Females accounted for 67% of patients in the dataset. Two percent of the patients lived outside of the five New York City boroughs, with 33% living in Queens, 25% living in Brooklyn, 23% living in Manhattan, and 17% living in the Bronx.

### What predicts no-shows?

**Fig. 1:**
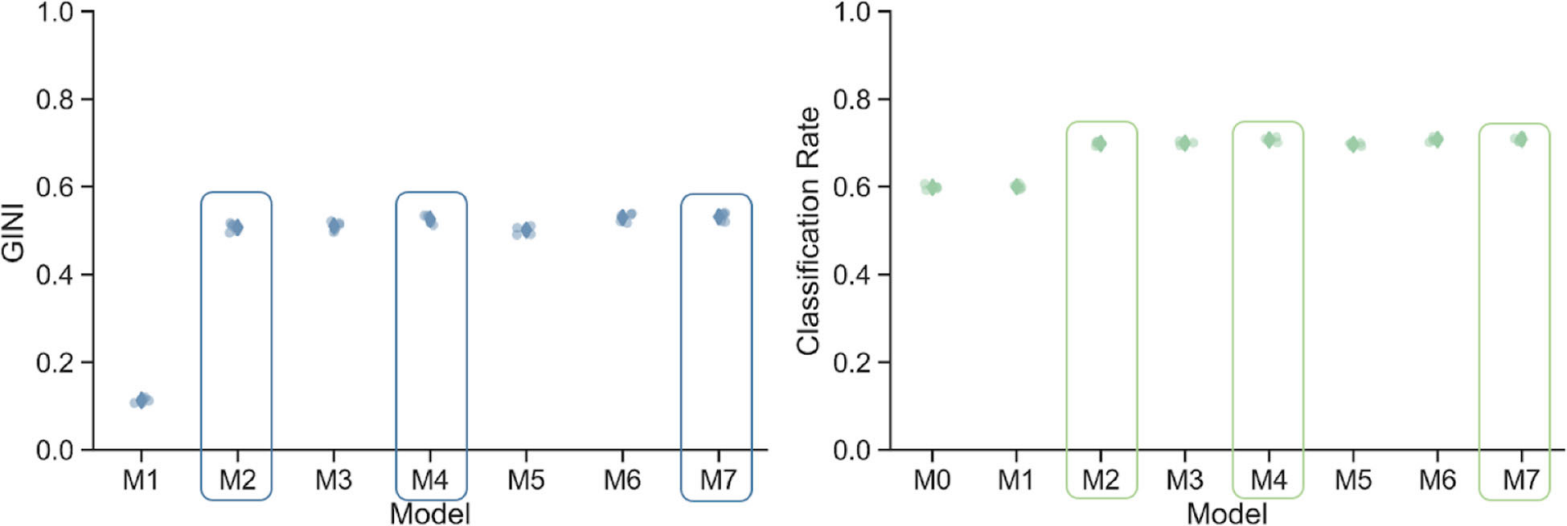
Cross-validated predictive performance of the adaptive models. Transparent circles show the performance of each fold, Solid diamonds show the mean performance across all 5 folds

This study has identified predictors of no-shows as a function of the borough of patient residence, the healthcare center they attended, empaneled provider, and lead time, while accounting for (and testing the extent of) variations between health centers. To test these, seven statistical models of increasing complexity were compared against a null-model that predicted that every person would attend their appointment (see Fig. 1).

**Fig. 2:**
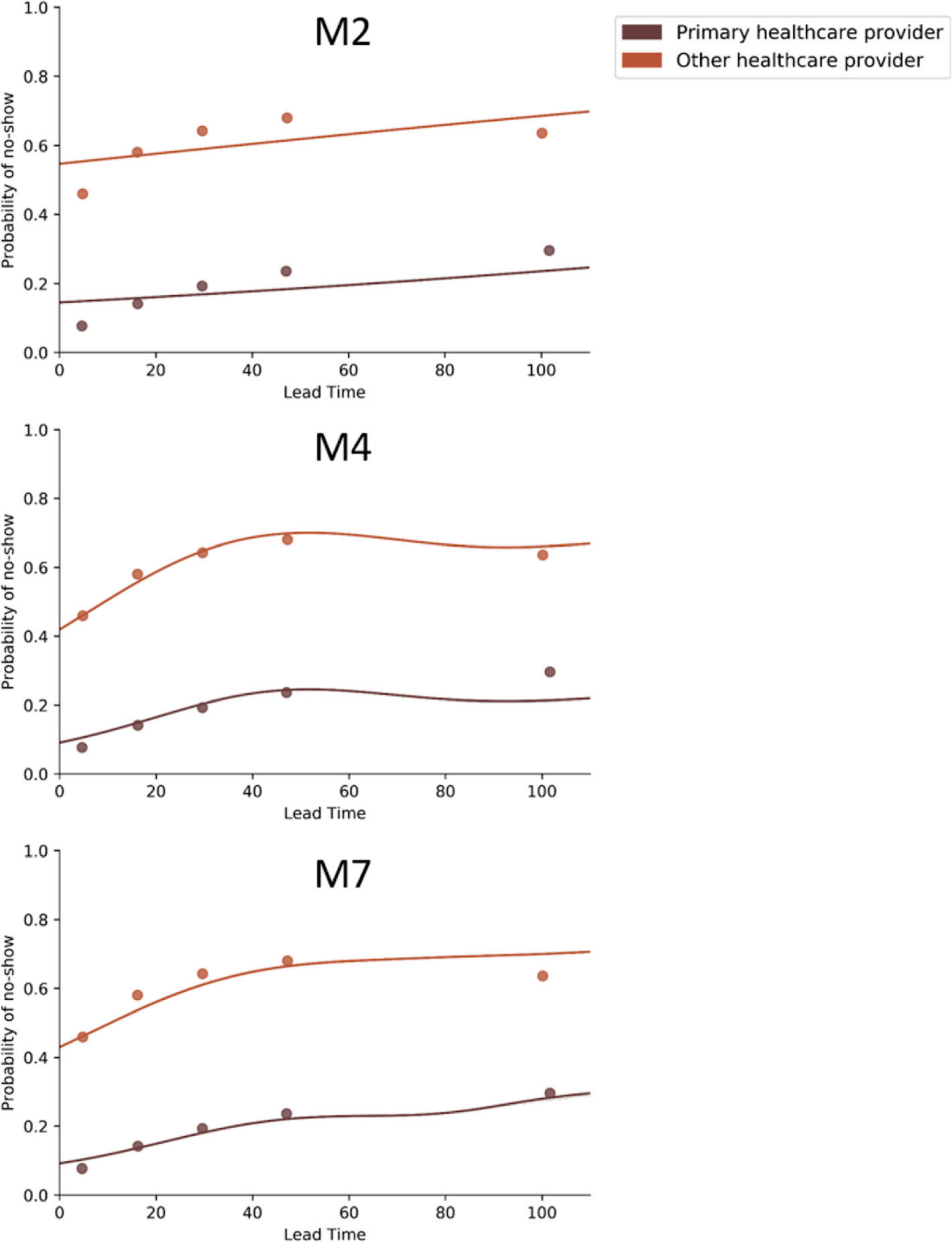
Non-linear effects of lead time on no-show rates. Dots are empirical quintiles based on lead time. Lines are model predictions

**Fig. 3:**
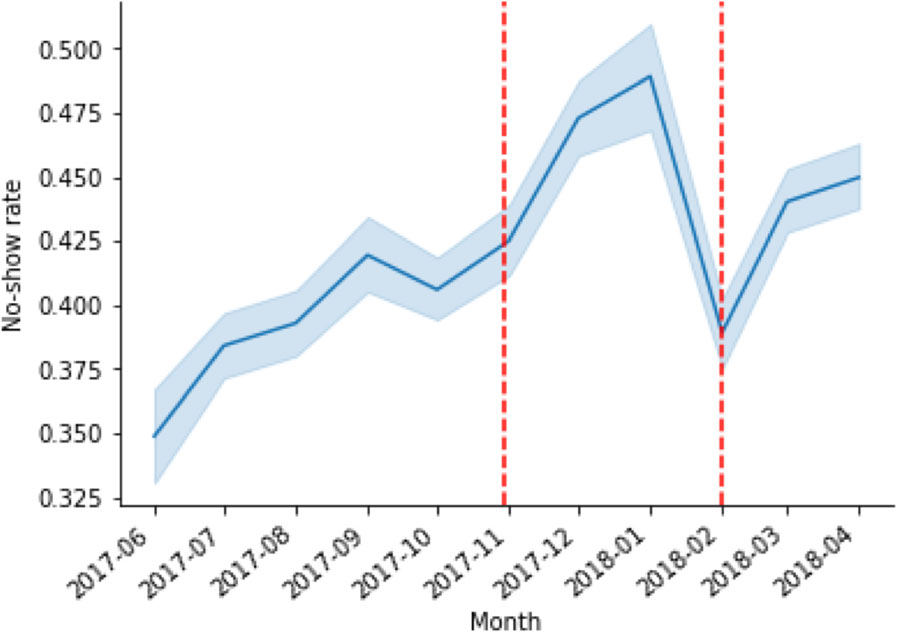
Aggregate no-show rate over time. The red lines indicate two stages of implementation of the reminder

The null-model will always predict the most common outcome, in this case attendance. Model 1 captures geographic variations in outcome by allowing the intercept to vary by health center and borough of residence. Model 2 introduces lead time and Empaneled provider as fixed-effects. This means that the model assumes that lead time and empaneled provider influence the no-show rate the same way across health centers. Models 3 through 7 built on Model 2 by allowing the effects of the predictors to vary by health center and borough and allowing lead time to have non-linear effects. Two of the nonlinear models, Model 4 and 7, were found to yield small but reliable improvements in fit, as can be seen in Fig. 3. Model 4 permits lead time to have the same nonlinear effect across all facilities and boroughs. Model 7 allows lead time to have different nonlinear effects in different facilities without any constraints on variations between facilities. Figure 1 compares the cross-validated model performance in terms of GINI and classification rate, Fig. 2 plots the model predictions of Models 2, 4, and 7 in relation to the quintiles of the data across all clinics.

Model 7 outperformed model 4 when predicting visits to empaneled providers following long lead times. However, Model 7 makes slightly worse predictions for visits with long lead times to non-empaneled providers. Moreover, while Model 7 outperforms Model 4 per facility, it only did so slightly. This suggests that the effect of lead-time is fairly stable between facilities.

The predictive modeling determined that the strongest predictor of no-show rates in FQHCs is empaneled provider, followed by lead time. To illustrate, in Model 2 (the first model that included both predictors) empaneled provider (z = − 91.45, *p* < 10^− 10^) had an unsigned z-value more than 4 times that of lead time (z = 23.87, p < 10^− 10^). The relative effect of the two predictors hold when the linearity assumption is relaxed, see Fig. 2. While these two variables were the strongest predictors of no-show behavior, there is no evidence to conclude that they interact with each other. Furthermore, the effects of empaneled providers did not differ between facilities. The length of lead time in days did affect each facility differently; but accounting for these differences only led to a small increase in predictive performance.

### Effectiveness of reminder intervention

There was no significant effect of the reminder on no-show rates. The no-show rate for appointments prior to the reminder was 41.6, and 42.1% after the reminder was implemented. For each individual facility location, changes in no-show rates ranged from a decrease of 3.0% to an increase of 5.6%. For a full overview of the changes in no-show rates across the facilities, see Table 2. For the 5569 individuals that had appointments both before and after the implementation of the reminder, the no-show rate declined significantly from 47.6 to 45.6%, χ^*2*^ = 5.7, *p < 0.05*. Finally, we ran a hierarchical generalized linear model to test whether the reminder was particularly effective for any specific lead time. No significant interaction between lead time and the reminder in predicting no-show rates was found (z = 0.39, *p* = .69), suggesting that the reminders were ineffective across lead times.

**Table 2:** No-show rates before and after the reminder intervention for each facility

Facility	No-show rate before reminder	No-show rate after reminder	Difference
1	42.56%	47.10%	−4.54%
2	45.05%	42.61%	+ 2.44%
3	41.49%	39.87%	+ 1.62%
4	37.51%	34.55%	+ 2.97%
5	37.68%	37.25%	+ 0.44%
6	43.38%	48.98%	−5.60%
7	42.52%	45.20%	−2.68%
8	46.28%	45.44%	+ 0.84%
9	34.35%	38.13%	−3.78%
10	44.53%	43.08%	+ 1.45%
11	47.68%	50.68%	−3.00%

Figure 3 indicates the aggregate no-show rates across time, before and after the reminder. The red lines indicate the moments where the reminder was implemented, with two facilities implementing the reminders in November 2017, and nine facilities implementing the reminder in February 2018. Weekly no-show rates were volatile throughout the trial (see Fig. 3), with rates ranging from 32 to 54% (σ *= 4,5%*).

## Discussion

The purpose of this study was to gain more knowledge on no-show behavior among vulnerable populations, served in urban FQHC settings. The analysis centered on two main aspects: identifying predictors of no-shows, and an analysis of the effectiveness of a reminder. These aspects provide valuable insights for future interventions that aim to decrease no-show at preventive appointments, which is important for clinical management and public health. From the clinical perspective, better precision in anticipating no-show offers better resource allocation and staff management [16]. From a public health perspective, a greater understanding of the barriers to attend increases the probability of introducing effective interventions that improve access to care, which should improve health outcomes, particularly in disadvantaged populations [17].

The two strongest predictors of no-shows identified in our analyses are seeing an empaneled provider and the lead time for making the appointment. While perhaps underwhelming, these are important levers to address. First, the ability to see a regular, familiar primary care provider appears to be critical. This makes the care received more personal, and can lower the emotional barrier to care. This is important as fear [18] and a lack of trust towards providers [19] reduce the likeliness to show, especially among vulnerable populations. The impact of empaneled providers on reducing emotional barriers should be explored further. The second lever is to reduce the lead time between the initial booking and the appointment. This way the health issue remains pressing, and it provides a sense of urgency and importance towards the patient [18]. These results are generalizable to other settings, as location effects did not influence the importance of empaneled provider, and it did only slightly for lead time. On top of reducing no-show rates, addressing these levers will benefit quality of care and patient satisfaction.

In addition, this study analyzed the effect of an increasingly popular behavioral intervention to decrease no-show: a reminder [12]. The ease of implementing this “nudge” can explain its wide use [20]. However, in our low-income setting, the reminder had no effect on no-show rates. Our finding of its ineffectiveness may caution its widespread adoption.

For patients with appointments before and after the implementation of the reminder, the no-show rate declined by 2 %, and may be more a result of attending because they no-showed previously, rather than because of an intervention. This is a limited and small result, especially given the persistent no-show rate and high potential for improvement in this setting. As only a small portion of studies on reminders have resulted in positive effects [20], this raises the question of whether a reminder in itself is ineffective, or whether it is certain settings or circumstances that inhibit the effect.

As underserved populations face a multitude of barriers to care, interventions targeted towards one barrier will not be sufficient, even when the intensity of the intervention offered is high. In Philadelphia, free transportation was offered to low-income urban patients, in response to concerns that transportation is a significant barrier to care [11]. Even though this approach had much more potential than a simple reminder, it had no effect on no-show rates [11]. Instead of discarding these interventions altogether, we argue that optimal outcomes can be reached when these interventions are targeted towards those in need. In that regard, reminders may still be useful when targeted towards patients that experienced extensive lead times for appointments (i.e., greater than 30 days) or that do not face additional barriers to care. In this way, perhaps the Philadelphia trial might have generated optimal results if transportation was only offered to individuals that explicitly noted a lack of transportation. Moving beyond a one-size fits all approach is an important concern for future research.

This is particularly helpful to address complex issues such as no-shows among vulnerable populations, as interventions need to be carefully targeted and combined. This requires careful analysis of barriers and more extensive data collection, and will lead to either higher administrative workloads or computational programs and skills to automatically target these interventions to those in need. As smart targeting techniques are increasingly available and refined, it will not be long before these can be widely applied, and we can start to address persistent barriers to care for underserved communities, while also considering the financial implications for doing so.

While there will always be no-shows – by choice, by error, or by obstruction – there is ample room for improvement. Due to the large amount of direct costs and lost revenue caused by each missed appointment, there is still tremendous potential for interventions to decrease no-show rates [21]. For this particular dataset, we estimated direct costs to the clinic at $29.82 and loss of revenue at $89 per appointment missed (see Appendix B). These estimates are applicable only to this individual network and therefore have not been explored in detail in the main text, as it is unclear how relevant these values would be to other FQHCs, even in urban areas. However, having such values can be especially useful, because it sets a reference point for determining necessary investment levels for interventions.

### Implications

This study provide important theoretical and practical insights. In terms of theory, the primary implication is that standard behavioral interventions are likely to be less effective for disadvantaged populations, given the additional barriers faced by these individuals. More study on this would be valuable as it has clear links to application for both policy and clinical practice.

The absence of an interaction between lead time and empaneled provider indicates that composite interventions may not necessarily yield greater net effects in these groups, but instead, should be tailored to individual needs, to the extent possible. From a policy and clinical practice perspective, this would add some effort to implementing interventions that work in such heterogeneous communities, but increasing attendance in these populations will have both immediate and long-term financial benefits as well as improving community health and well-being.

These insights apply to preventive care appointments, as treatment and urgent care would have a number of different factors in terms of behavior based on structural and psychological barriers. However, the general concept of limited effects for disadvantaged populations remains relevant.

### Limitations

The over dispersion and volatility of data from this population are likely to mask underlying effects, as is evident in the varying clinical rate changes before and after the reminder intervention and across different facilities. It is possible that broad applications of interventions such as reminders have uneven effects for different populations, that could mitigate when reviewed in composite. Why the intervention seemed effective across some facilities and not in others has not been investigated further as there was no data on potential contributing factors available at the time of this study. Future work will need to identify specific areas of impact, while also indicating low-return groups where other interventions may be more likely to be effective. This will provide valuable insights for future targeting of interventions.

## Conclusions

This study identified meaningful insights on addressing no-shows from two perspectives. The first perspective is clinical management: overbooking has typically been the approach used to address no-shows, yet understanding predictors of no-shows as presented here can recalibrate interventions to address the underlying behavior (or barrier) instead, and to align choice architecture with attending. Concurrently, the public health perspective of no-shows is an equally critical second aspect, as patients in FQHCs are typically from high-risk, vulnerable populations. This implies that their likelihood of subsequent illness or hospitalization is high, yet possible to avoid if receiving preventive care.

Through prediction models, we identified two important levers that appear to have direct influence on no-shows. The first is specifically ensuring that patients see their primary care provider whenever possible. The second is to reduce the lead time between the initial booking and the appointment. The limited effects of the reminder suggest the need for more personalized behavioral interventions to reduce no-shows. Together, these findings provide a starting point for future interventions to decrease no-show rates through more contextually appropriate methods, and in doing so improving the health outcomes of underserved communities.

## Figures and Tables

**Fig. 4:**
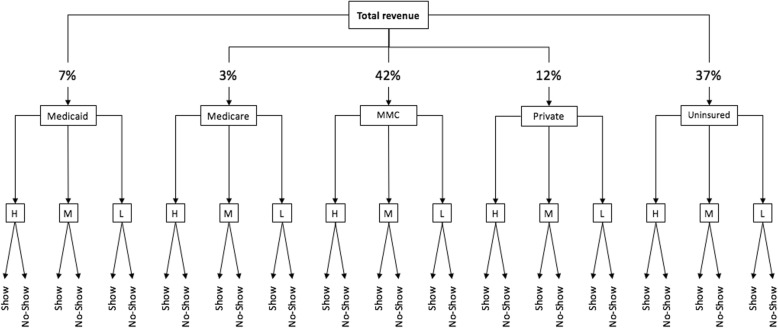
Decision tree figure

## Data Availability

The data is protected under HIPAA security regulation and cannot be shared.

## Appendix A

### Statistical Methods for predictive models

The aim of this analysis was to evaluate the functional form of the effect of lead time and to test its relationship with empaneled providers, while respecting the hierarchical structure of the data, as patients were nested inside clinics, and came from different boroughs in New York City. This was achieved by fitting a set of hierarchical generalized linear models, and generalized additive models and comparing their out-of-sample predictive accuracy (OSSPA) with five-fold cross validation. Two measures of OSSPA, classification rate and GINI coefficients were used. GINI coefficients are a signal-theoretic measure that captures how well a model can discriminate between two options, where chance performance gives a coefficient of 0 and perfect discrimination gives a coefficient of 1, it is directly linked to AUROC so that GINI = 2*AUROC – 1. Analyses were conducted in R (v 3.5.2.). Generalized linear models were fitted using the lme4 (v 1.1–20) package, generalized additive models were fitted with mgcv (v 1.8–26). Before analyzing the data, patients who lived outside of NYC were excluded, as did patients from Staten Island, as there were too few of them to get reliable coefficient estimates.

The eight fitted models were as follows:

*Logistic Models.*

**M0:** Predicting no show status from global intercept only.

**M1:** Predicting no show status for random intercepts for healthcare facility and random intercepts for borough.

**M2:** Retains the random intercepts and adds linear fixed effects for lead time and empaneled provider.

**M3:** Retains the random intercepts and adds random effects for lead time and empaneled provider (varying both by healthcare facility and borough).

M1 provided a modest but reliable improvement in OSSPA compared to M0, inspection of the variance decomposition suggested that this improvement was almost entirely driven by variation in intercepts by healthcare facilities. M2 provided a large improvement relative to M1, but M3 had slightly lower OSSPA relative to M2. In other words, the linear models provided strong evidence that both lead time and empaneled provider predicted no-show, but no evidence that these effects differed between health facilities or boroughs, and the general weight of evidence suggested that the home borough of patients did not meaningfully predict no-show. For this reason, we focused on exploring non-linear effects based on health-care facilities (though we retained random intercepts for boroughs, to ensure comparability with the generalized linear models).

*Generalized Additive Models.*

**M4:** As M2 but rather than assuming a linear effect for lead time, the effect of lead time was estimated with a thin plate spline.

**M5:** As M4 but lead time was now allowed to interact with empaneled provider, testing whether the effect of lead time was influenced by provider type.

**M6:** As M4 but allowing lead time to vary by medical facility, with a shrinkage parameter that limits between-facility variation of the mathematical function. This is conceptually similar to a random effect in the glm framework, where variation between clusters are allowed, but are constrained by a general trend.

**M7:** As M6 but without a shrinkage parameter. Meaning that the mathematical function of lead time was independently fitted for each facility.

Though the raw data for these analyses cannot be shared, because of patient privacy concerns, the annotated r-code of these analyses are supplied below, to allow other public health researchers to run similar analyses, or extend this paradigm to other datasets and problems.

# #Rcode and results for predictive models and five-fold cross-validation are in the same typeface here. This should be noted if testing reproducibility.

### Loading packages.

library(caret).

library(glmnet).

library(pROC).

library(lme4).

library(mgcv).

### Session info.

sessionInfo().

R version 3.6.1 (2019-07-05).

Platform: x86_64-w64-mingw32/× 64 (64-bit).

Running under: Windows 10 × 64 (build 17,763).

Matrix products: default.

locale:

[1] LC_COLLATE = English_United Kingdom.1252.

[2] LC_CTYPE = English_United Kingdom.1252.

[3] LC_MONETARY = English_United Kingdom.1252.

[4] LC_NUMERIC=C.

[5] LC_TIME = English_United Kingdom.1252.

attached base packages:

[1] tools stats graphics grDevices utils datasets methods.

[8] base

other attached packages:

[1] lme4_1.1–21 pROC_1.15.3 glmnet_2.0–16 foreach_1.4.4.

[5] Matrix_1.2–17 caret_6.0–83 ggplot2_3.1.1 lattice_0.20–38.

[9] R.utils_2.8.0 R.oo_1.22.0 R.methodsS3_1.7.1 mgcv_1.8–26.

loaded via a namespace (and not attached):

[1] Rcpp_1.0.1 nloptr_1.2.1 pillar_1.3.1 compiler_3.6.1.

[5] gower_0.2.0 plyr_1.8.4 iterators_1.0.10 class_7.3–15.

[9] boot_1.3–20 rpart_4.1–15 ipred_0.9–8 lubridate_1.7.4.

[12] tibble_2.1.1 gtable_0.3.0 nlme_3.1–139 pkgconfig_2.0.2.

[16] rlang_0.3.4 prodlim_2018.04.18 stringr_1.4.0 withr_2.1.2.

[20] dplyr_0.8.0.1 generics_0.0.2 recipes_0.1.5 stats4_3.6.1.

[25] nnet_7.3–12 grid_3.6.1 tidyselect_0.2.5 data.table_1.12.2.

[29] glue_1.3.1 R6_2.4.0 survival_2.44–1.1 minqa_1.2.4.

[33] lava_1.6.5 reshape2_1.4.3 purrr_0.3.2 magrittr_1.5.

[37] ModelMetrics_1.2.2 splines_3.6.1 MASS_7.3–51.3 scales_1.0.0.

[41] codetools_0.2–16 assertthat_0.2.1 timeDate_3043.102 colorspace_1.4–1.

[45] stringi_1.4.3 lazyeval_0.2.2 munsell_0.5.0 crayon_1.3.4.

### Setting seed to ensure reproducibility.

set.seed(278).

### Creating the folds.

Folds = groupKFold(rownames(df), k = 5).

### Specifying the generalised linear models.

Model_0 = “NoShow ~ 1” #Null model, predicting no-shows from global intercept.

Model_1 = “NoShow ~ 1 + (1|Bourough) + (1|Facility)” #Hierarchical null model, allow intercept to vary by borough and facility.

Model_2 = “NoShow ~ 1 + Visit_with_empanel_prov + lead_time + (1|Bourough) + (1|Facility)” # Introduces empanelled provider and lead time as fixed effect predictors.

Model_3 = “NoShow ~ 1 + Visit_with_empanel_prov + lead_time + (Visit_with_empanel_prov + lead_time|Bourough) + (Visit_with_empanel_prov + lead_time|Facility)” # Allows empanelled provider and lead time to vary by facility and borough.

### Fitting the generalised linear models.

fit_list = NULL.

gini_list = NULL.

sensitivity_list = NULL.

specificity_list = NULL.

accuracy_list = NULL.

for (i in 1:5){.

### Specify the training and test subset for this fold.

Training = unlist(Folds[i], use.names = F).

Test = setdiff(rownames(df), Training).

### Fit the model on the training data.

fit = glm(Model_0, data = df[Training,], family = “binomial”) # Replace Model_0 with the desired model.

### Store the fit object for later inspection.

fit_list = append(fit_list, fit).

### Evaluating the OOS Performance of the model, and store the metrics.

OOS_Y = as.numeric(df[Test, “NoShow”]).

OOS_PRED = rep(round(mean(df[Training, “NoShow”])), length(df[Test, “NoShow”])).

OOS_roccurve = roc(OOS_Y ~ OOS_PRED).

OOS_gini = 2*auc(OOS_roccurve)-1.

gini_list = append(gini_list, OOS_gini).

OOS_sensitivity = mean(OOS_Y[OOS_Y==1] == OOS_PRED[OOS_Y==1]).

sensitivity_list = append(sensitivity_list, OOS_sensitivity).

OOS_specificity = mean(OOS_Y[OOS_Y==0] == OOS_PRED[OOS_Y==0]).

specificity_list = append(specificity_list, OOS_specificity).

OOS_accuracy = mean(OOS_Y == OOS_PRED).

accuracy_list = append(accuracy_list, OOS_accuracy).

}

# Specifying the generalised additive models.

Model_4 = “NoShow ~ Visit_with_empanel_prov + s(lead_time, bs=‘tp’) + s(Bourough, bs=‘re’) + s(Facility, bs=‘re’)” # Allows lead_time to have a fixed non-linear effect.

Model_5 = “NoShow ~ Visit_with_empanel_prov + s(lead_time, by=Visit_with_empanel_prov, bs=‘tp’) + s(Bourough, bs=‘re’) + s(Facility, bs=‘re’)” #Includes a fixed-effects interaction term between lead time and empanelled provider (as well as main effects).

Model_6 = “NoShow ~ Visit_with_empanel_prov + s(lead_time, bs=‘tp’) + s(Bourough, bs=‘re’) + s(lead_time, Facility, bs=‘fs’)” #Includes a non-linear random effect for lead_time, and a fixed effect for empanelled provider, as well as random intercepts for borough and facility.

Model_7 = “NoShow ~ Visit_with_empanel_prov + s(lead_time, by=Facility, bs=‘tp’) + s(Bourough, bs=‘re’) + s(Facility, bs=‘re’)” #Includes a non-linear random effect for lead_time, that is unconstrained by hyper parameters (i.e. there is no assumption that the effect of lead time is consistent in any way across facilities), and a fixed effect for empanelled provider, as well as random intercepts for borough and facility.

### Fitting the generalised additive models.

fit_list = NULL.

gini_list = NULL.

sensitivity_list = NULL.

specificity_list = NULL.

accuracy_list = NULL.

for (i in 1:5){.

### Specify the training and test subset for this fold.

Training = unlist(Folds[i], use.names = F).

Test = setdiff(rownames(df), Training).

### Fit the model on the training data.

fit = gam(Model_4, data = df[Training,], method = “REML”,family = “binomial”) #Add models.

### Store the fit object for later inspection.

fit_list = append(fit_list, fit).

### Evaluating the OOS Performance of the model, and store the metrics.

OOS_Y = as.numeric(df[Test, “NoShow”]).

new = df[Test, c(“Bourough”, “Facility”, “lead_time”, “Visit_with_empanel_prov”)].

OOS_PRED = predict(fit, type = ‘response’, newdata = new).

OOS_roccurve = roc(OOS_Y ~ OOS_PRED).

OOS_gini = 2*auc(OOS_roccurve)-1.

gini_list = append(gini_list, OOS_gini).

OOS_sensitivity = mean(OOS_Y[OOS_Y==1] == round(OOS_PRED[OOS_Y==1])).

sensitivity_list = append(sensitivity_list, OOS_sensitivity).

OOS_specificity = mean(OOS_Y[OOS_Y==0] == round(OOS_PRED[OOS_Y==0])).

specificity_list = append(specificity_list, OOS_specificity).

OOS_accuracy = mean(OOS_Y == round(OOS_PRED)).

accuracy_list = append(accuracy_list, OOS_accuracy).

}

## Appendix B

### Cost analysis

To build a complete cost profile, five variables were included in the model: (a) costs incurred and payments received per encounter, (b) no-show status per appointment, appointment length, (c) payer types, and (d) visit dates. Hourly wages paid to staff members in clinics were used to estimate the staff costs associated with each appointment.

To quantify the financial impact and present the potential cost savings of reducing no-shows in this FQHC setting, available cost data and patient information on the 63,842 individuals were analyzed to retrieve precise estimates. The cost profile was set up based on an itemization of particular cost factors combined with assumptions on how costs should be derived. A decision tree model (see Fig. 4), which allows for reconstruction of scenarios based on payer category *(Medicaid, Medicare, Medicaid Managed Care (MMC), Private Insurance and Uninsured*), appointment type (*high (H), medium (M)*or *low (L)*cost) and no-show status (*Show or No-show*), was developed. The decision tree model incorporates probabilities and costs for various scenarios.

To quantify the financial impact of no-shows on the healthcare clinic and present the impact on total revenues after decreasing no-show rates, reductions of no-shows in the given setting were simulated, and the revenues for attended encounters (shows) (Rae) and no-shows(Rns) were calculated based on the decision tree model. The following equations were used respectively, including one additional variable (*Cns*) to account for the costs incurred in the case of a no-show:

*Rae = (PP + NP–C)*X.*

*Rns = (PP + NP – (C + Cns))*X.*

*Rae* = Revenue for attended encounters, Rns = Revenue for no-show, PP = Prospective Payment, NP = Net Payment, C = staff costs, X = number of appointments.

Figure: Decision tree model on costs associated with no-shows across various appointment and payer categories. Categories are mutually exclusive total numbers sum to 101 because of rounding errors.

*The five most common payment types for the participating health centers are Medicaid, Medicare, Medicaid Managed Care (MMC), Private Insurance and Uninsured/Self-Pay. MMC represents various insurance arrangements, combined here for simplicity. Appointment types H, M and L (high/medium/low) occur with probabilities of 20, 53 and 27% in each payer type. Show and no-show probabilities are 56.93 and 43.07% for each appointment type.*

In order to account for inaccuracies in the estimation of cost values, probabilistic and deterministic sensitivity analyses were conducted. All analyses and simulations were run in R.

*Cost Analysis.*

FQHCs operate under a unique reimbursement scheme, allowing them to provide services to patients regardless of their ability to pay. Depending on the payer category (Medicaid, Medicare, Medicaid Managed Care (MMC), Private Insurance), the clinic receives different payments, including a flat payment rate per visit. Uninsured patients without the ability to pay are covered through federal grants, received annually by the health center. The costs per appointment differ depending on the types of appointments, the length of the appointment, as well as the specialty of the physician. Our findings suggest that the marginal cost of a no-show at an FQHC, considering all payer and appointment types, is $29.82, and the average revenue per patient is $89.66.

## Abbreviations

FQHCs: Federally Qualified Health Centers
AUROC: Area Under the Receiver Operating Characteristics
